# MICRORNA BIOMARKERS AND POTENTIAL MECHANISMS ASSOCIATED WITH VO2PEAK VARIATION IN OLDER ADULTS

**DOI:** 10.1093/geroni/igae098.3673

**Published:** 2024-12-31

**Authors:** Genesio Karere, Fang-Chi Hsu, Peggy Cawthon, Xiping Zhang, Karyn Esser, Laura Cox, Stephen Kritchevsky

**Affiliations:** Wake Forest University School of Medicine, Winston-Salem, North Carolina, United States; Wake Forest University School of Medicine, Winston-Salem, North Carolina, United States; California Pacific Medical Center, San Francisco, California, United States; University of Florida, University of Florida, Florida, United States; University of Florida, Gainesville, Florida, United States; Wake Forest University School of Medicine, WINSTON-SALEM, North Carolina, United States; Wake Forest University School of Medicine, Wake Forest University School of Medicine, North Carolina, United States

## Abstract

Peak oxygen consumption during exercise (VO2peak) is an indicator of cardiorespiratory and overall fitness. However, some older adults are intolerant to exercise, impeding VO2peak measurement and necessitating development of alternative methods to screen patients for VO2peak and define pathways that accompany VO2peak variation. We used RNA sequencing to identify expressed microRNAs (miRNAs) and mRNAs in serum and skeletal muscle from 72 participants (70-91 years old; females=53%) in the Study of Muscle, Mobility and Aging, with low (11-14.7 ml/kg/min) or high (28.3-39.4) VO2peak (N=18/group) as well as 36 random samples spanning the entire spectrum of VO2peak. We analyzed samples discordant for VO2peak using the Linear Models for Microarray/RNAseq Data analysis package and identified five serum miRNAs (miR-1301-3p, -431-5p, -501-5p, -519a-3p, and -18a-3p) that discriminate individuals with low or high VO2peak (adjusted p< 0.05). In muscle, 14 miRNAs also displayed differential expression in low and high VO2peak samples. We identified sex differences in expression of 8 miRNAs in muscle (adjusted p< 0.05). Ten differentially expressed (DE) miRNAs, predicted to regulate 50 DE miRNAs, showed an inverse expression pattern in muscle, consistent with direct participation in gene regulation and VO2peak variation. We used Weighted Gene Co-expression Network Analysis and Ingenuity Pathway Analysis tools and identified ME Cyan as the top-most gene module correlated with VO2peak (r=0.7, p=4e-12). Pathways enriched with the module genes included nitric oxide synthesis and signaling. In conclusion, we identified a panel of potential circulating and tissue miRNA biomarkers of VO2peak in older adults and potential pathways that associate with VO2peak variation.

